# Differences in morphological and physiological features of citrus seedlings are related to Mg transport from the parent to branch organs

**DOI:** 10.1186/s12870-021-03028-z

**Published:** 2021-05-27

**Authors:** Yamin Jia, Hao Xu, Yuwen Wang, Xin Ye, Ningwei Lai, Zengrong Huang, Lintong Yang, Yan Li, Li-Song Chen, Jiuxin Guo

**Affiliations:** 1 Fujian Provincial Key Laboratory of Soil Environmental Health and Regulation, College of Resources and Environment, Fujian Agriculture and Forestry University, Fuzhou, 350002 China; 2 International Magnesium Institute, College of Resources and Environment, Fujian Agriculture and Forestry University, Fuzhou, 350002 China

**Keywords:** Mg deficiency, Citrus plants, Mg uptake, Parent and branch properties, Morphological and physiological characteristics

## Abstract

**Background:**

In this study, we aimed to test the hypothesis that magnesium (Mg) remobilization in citrus plants is regulated by Mg supply and contributes to differences in the growth of the parent and branch organs. Citrus seedlings were grown in sand under Mg deficient (0 mmol Mg^2+^ L^−1^, -Mg) and Mg sufficient (2 mmol Mg^2+^ L^−1^, + Mg) conditions. The effects on biomass, Mg uptake and transport, gas exchange and chlorophyll fluorescence, as well as related morphological and physiological parameters were evaluated in different organs.

**Results:**

Mg deficiency significantly decreased plant biomass, with a decrease in total plant biomass of 39.6%, and a greater than twofold decrease in the branch organs compared with that of the parent organs. Reduced photosynthesis capacity was caused by a decreased in pigment levels and photosynthetic electron transport chain disruption, thus affecting non-structural carbohydrate accumulation and plant growth. However, the adaptive responses of branch leaves to Mg deficiency were greater than those in parent leaves. Mg deficiency inhibited plant Mg uptake but enhanced Mg remobilization from parent to branch organs, thus changing related growth variables and physiological parameters, including protein synthesis and antioxidant enzyme activity. Moreover, in the principal components analysis, these variations were highly clustered in both the upper and lower parent leaves, but highly separated in branch leaves under the different Mg conditions.

**Conclusions:**

Mg deficiency inhibits the growth of the parent and branch organs of citrus plants, with high Mg mobility contributing to differences in physiological metabolism. These findings suggest that Mg management should be optimized for sustainable citrus production.

**Supplementary Information:**

The online version contains supplementary material available at 10.1186/s12870-021-03028-z.

## Background

Magnesium (Mg) is an essential element required for plant growth and development, playing important roles in many physiological and biochemical processes, including chlorophyll and protein synthesis, assimilate production and transport, and enzyme activities [1, 2]. It has been reported that up to 35% of the total Mg in plants is bound to chlorophyll molecules, in which Mg is the central atom and determines its structure and function, further affecting photosynthesis, carbohydrate accumulation, energy metabolism, and stress resistance [2–4]. Although Mg is an intermediate in many metabolic reactions, Mg deficiency is a common phenomenon in intensive agricultural production, severely affecting the yield and quality of various crops, including cereals, vegetables, and fruits [5, 6]. Dietary Mg intake is closely related to human health [7], with lower Mg intake from foods leading directly or indirectly to a series of human diseases, such as hypertension, diabetes mellitus, and myocardial infarction. Thus, a comprehensive understanding of the characteristics of Mg demand and the response to deficiency is of great significance in developing strategies for targeted Mg fertilizer management that improve the food yield and Mg nutrition quality of crops and human health [6–8].

Mg in plants is highly mobile, with Mg deficiency symptoms displayed preferentially in the lower or old leaves as a type of chlorosis (yellowing) between the veins [8, 9]. Although the effects of Mg deficiency on plant growth and nutritional function have been widely studied, the results have not always been consistent. Hermans et al. [10] reported that, while Mg deficiency induced a decrease in Mg concentration, the sugar content increased with rising leaf positions in sugar beet, and a clear negative correlation was identified between Mg concentration and sugar content. In contrast, Chen and Fan [11] reported that Mg deficiency was more marked in the upper or young leaves rather than in the lower or old leaves of banana plants. Moreover, the most marked damage was detected in the middle leaves, which exhibited marginal necrosis and wilting. Similar results were reported in soybean plants, suggesting that Mg mobility is related to its physiological and biochemical characteristics [2]. However, compared to other plants such as sugar beet [10, 12], wheat [13], and rice [14, 15], the effects of Mg deficiency on Mg mobility from parent to branch organs of woody plants, especially citrus plants, are not clear [5, 16].

Citrus (*Citrus reticulata* Blanco) is an evergreen woody fruit tree grown in tropical and subtropical regions, and makes a vital contribution to the economy and fruit production in many countries [17]. Citrus production in China [18–20], Brazil [21], India [22], and New Zealand [23], is commonly compromised by Mg deficiency caused by severe soil acidification, low soil quality, and excessive or imbalanced fertilizer usage. In addition, the large hydration radius of Mg^2+^ and its weak adsorption by soil colloids exacerbate the loss of Mg by leaching [8, 12]. Previous studies have indicated that citrus plants have a high demand for Mg, although this characteristics is often ignored in production. Mattos Jr. et al. [24] reported that Mg demand of a 6-year-old sweet orange tree was 8.7 kg ha^−1^, which is comparable to the demand for phosphorous (P, 8.3 kg ha^−1^). In nutrient uptake assessment, approximately 43 kg Mg ha^−1^ was measured in the above-ground organs of a mature orange tree, with an apparently higher annual Mg uptake balance (12.6 kg ha^−1^) compared with that of P (8.6 kg ha^−1^) [25]. These findings suggested that Mg management may be helpful in improving plant growth as well as fruit yield and quality in citrus production [19, 21, 22, 26]. Similar results were also observed in citrus seedlings, with greater effects of Mg deficiency on lower leaves compared with those on the upper leaves. These findings indicated that the effect of Mg deficiency in plants is related to differences in the Mg concentration caused by changes in Mg mobility [9, 27]. Therefore, understanding the Mg transport mechanisms involved in the response of both parent and branch organs of a citrus plant to Mg deficiency is of great theoretical and practical significance for intensive agricultural production with high yield and high quality.

In this study, we investigated the effects of Mg deficiency on biomass, Mg uptake, gas exchange, chlorophyll fluorescence transients, and related morphological and physiological parameters in different organs of citrus seedlings. We also focused on the impact of Mg deficiency on Mg transport capacity from the parent to branch organs.

## Methods

### Plant materials and growing conditions

The citrus plant ‘Xuegan’ (*Citrus sinensis* (L.) Osbeck) was used in this study, which fresh fruits collected from the Fujian Academy of Forestry Sciences (Fuzhou, China). The experimental conditions for the control and Mg deficiency were designed according to those reported by Cai et al. [28] and Ye et al. [9]. Briefly, citrus seedlings were cultivated under greenhouse conditions with a natural photoperiod in Fuzhou city, China (annual mean sunshine 1,700–1,980 h and annual mean temperature 20 ºC–25 °C). After seed germination and seedling establishment in the seedling-raising plate, two similar seedlings were transplanted to a 6 L ceramic pot (26 cm top diameter × 16 cm bottom diameter × 22 cm in height) containing 5 kg clean sand, with 20 repeats or pots for each treatment. The seedlings were cultivated to the stage with an average of three leaves (1 g fresh weight and 10 cm height). The seedlings were treated with modified Hoagland’s nutrient solution to provide the Mg treatments conditions for Mg deficiency (0 mmol L^−1^ Mg(NO_3_)_2_, -Mg) and Mg sufficiency (2 mmol L^−1^ Mg(NO_3_)_2_, + Mg). The composition of other nutrients was as follows: 5 Ca(NO_3_)_2_, 2 K_2_SO_4_, 1 KH_2_PO_4_, 1 KNO_3_ in macronutrients (mmol L^−1^), and 20 Fe-EDTA, 2 MnCl_2_·4H_2_O, 0.5 CuSO_4_·5H_2_O, 2 ZnSO_4_·7H_2_O, 10 H_3_BO_3_, and 0.065 (NH_4_)_6_Mo_7_O_24_·4H_2_O in micronutrients (μmol L^−1^). The N content in the Mg deficiency treatment group was compensated by the addition of NH_4_NO_3_. Seedlings were irrigated every 2 days with nutrient solutions, and the pH regulated at 6.50 ± 0.05. Intermittent drip irrigation of 0.5 L tap water containing 1.7 mg L^−1^ Mg^2+^ was applied to the sand surface to avoid drying. After 11 months of culture from May 2018 to April 2019, plant samples were collected and determined for morphological and physiological characteristics, in which the parameters both gas exchange and fluorescence transient were measured early in living plants (March 2019).

### Sampling and biomass measurements

To understand the effect of Mg on branch growth and the transport capacity of Mg from parent to branch, citrus seedlings were harvested on a sunny day, with one plant in each pot was used for biomass measurements and the other was used for other assays. For plant biomass measurement, citrus seedlings were separated into root, stem, and leaf organs. The root system was further divided into the main root (MR) and lateral root (LR), and the stem was divided into branch stem (BS) and parent stem (PS). The PS was subdivided into the upper stem (US) and lower stem (LS). The leaves were divided into branch leaves (BL) and parent leaves (PL). The PL was subdivided into the upper leaves (UL) and lower leaves (LL). The US and UL or LS and LL were defined based on 1/2 plant height, the MR and LR were defined as > 1 mm and < 1 mm root diameter, respectively. Some citrus seedlings were selected for collection of fresh samples. The intermediate leaves (2/5 and 4/5 plant height and 1/2 branch length) of the stem and branches and the white active root (0.5–1.0 cm from the root tip) were sampled for physiological and biochemical measurements. Some fresh samples were also collected during 10:00 and 12:00 on a sunny day and frozen in liquid N_2_ and then stored at –80 °C.

Biomass dry weight was measured after drying samples at 105 °C for 30 min and storage at 70 °C for approximately 2 days until a constant weight was achieved. The root/shoot, stem cross area (πr^2^, where r = 1/2 stem base diameter), and biomass distribution (organ or part biomass/whole plant biomass × 100%) was calculated. The plant height, number of plant leaves, first branch height, number of branches, branch length, and number of branch leaves were recorded.

### Leaf growth characteristics and root morphological measurements

The characteristics of leaf growth, including fresh weight, dry weight, water content ([fresh weight—dry weight]/fresh weight × 100%), leaf area, specific leaf weight (SLW, leaf dry weight/leaf area) and specific leaf area (SLA, leaf area/leaf dry weight) were calculated according to the methods described by Chen et al. [29]. Leaf area was determined by the combined application of the DR-6030C scanner (Canon, Beijing, China) and Image-Pro Plus 6.0 software (Media Cybernetics, Silver Spring, MD, USA).

The morphology parameters, including root length, surface area, average diameter, root volume, and the number of root tips were calculated. After the sand was washed from the fresh roots, images were captured using an Epson Expression 10000XL digital scanner (Epson, San Jose, CA, USA), and analyzed using WinRHIZO software (Version 2009b, Regent Instruments, Montreal, QC, Canada). The root activity was also measured using the tetrazolium chloride (TTC) staining method.

### Mg concentration, accumulation and distribution measurements

To determine the Mg concentration in different organs of citrus seedlings, the dried and ground samples were completely digested with HNO_3_-HClO_4_ at 250 ºC–300 ºC, and the absorbance was measured at 285.2 nm by inductively coupled plasma-optical emission spectroscopy (ICP-OES; Optima 7300 DV, PerkinElmer, MA, USA). The total Mg accumulation in the plant was calculated as the sum of the Mg accumulation in the organs (Mg concentration × dry weight in different organs). The Mg distribution in different organs or parts was calculated as: (Mg accumulation in different organs/the sum of total Mg accumulation in plants) × 100%.

### Soluble sugar and starch measurements

The soluble sugar and starch in the root and leaf were extracted and measured according to the method described by Ribeiro et al. [30]. Dried ground samples (0.1 g) were mixed with 10 ml 630 g L^−1^ ethanol and boiled for 30 min, then cooled and centrifuged for 5 min. The supernatant was collected and this extraction process was repeated twice. The ethanol in the supernatant was evaporated and the extract was reduced to 3 mL for soluble sugar analysis. The remaining residue was further extracted by the addition of 2 mL distilled water and boiling for 15 min. After the addition of 2 mL 9.2 mol L^−1^ HClO_4_ and cooling for 15 min, the mixture was centrifuged for 10 min. This extraction process was repeated twice and the supernatant was collected for starch analysis by the anthrone-ethyl acetate colorimetry method with absorbance at 630 nm measured using an ultraviolet–visible spectrophotometer (Libra S22, Biochrom Ltd, Cambridge, UK). The non-structural carbohydrate (NSC) content was calculated as the sum of soluble sugar and starch in each organ.

### Leaf pigments and gas exchange measurements

Citrus seedlings were cultivated under greenhouse conditions. Before the seedlings were harvested (March 2019), gas exchange in leaves was measured continuously between 9:00 and 11:00 on a sunny day using a portable photosynthesis system (CIARS-2, PP systems, Herts, UK). According to the method [9, 31], during the measurement, leaf temperature was controlled at 27 ± 0.5 °C and relative humidity was maintained at 45 ± 1% under a photosynthetic photon flux density (PPFD) of 1,000 μmol m^−2^ s^−1^. Finally, net photosynthetic rate (*P*_n_), stomatal conductance (*g*_s_), intercellular CO_2_ concentration (*C*_i_), transpiration rate (*T*_r_), and photosynthetic water-use efficiency (PWUE = *P*_n_ / *T*_r_) were measured.

Leaf chlorophyll (Chl) a, Chl b, and carotenoids (Car) was extracted from fresh samples with 80% (v/v) acetone and measured as described by Lichtenthaler and Wellburn [32]. The relative chlorophyll content of leaves was measured for determination of the chlorophyll index (SPAD) using a SPAD-502 Plus Chlorophyll meter (Minolta Camera, Osaka, Japan).

### Leaf Chl a fluorescence transients (OJIP) and related parameters measurements

After measuring gas exchange, the citrus seedlings were dark-adapted for approximately 3 h on the same day, and leaf Chl a fluorescence transients (OJIP) and related parameters were measured using a Handy Plant Efficiency Analyzer (Handy PEA, Hansatech Instruments Limited, Norfolk, UK). PPFD (3,000 μmol m^−2^ s^−1^) was provided by an array of three light-emitting diodes (650 nm). Based on the upper leaves of plants in the Mg supply group as the reference, we calculated the following Chl a fluorescence transient curves: V_t_ [*V*_t_ = (*F*_t_- *F*_o_)/(*F*_m_-*F*_o_), relative variable fluorescence at t between *F*_o_ and *F*_m_], W_k_ [*W*_k_ = (*F*_t_- *F*_o_)/(*F*_300µs_-*F*_o_), relative variable fluorescence at t between *F*_o_ and *F*_300μs_], and the ΔV_t_ (the differences of relative variable fluorescence at t between *F*_o_ and *F*_m_ reference control treatment) and ΔW_k_ (the differences of relative variable fluorescence at t between *F*_o_ and *F*_300μs_ reference control treatment). Also, the parameters including ΔK (the differences of relative variable fluorescence at t = 300 μs reference control treatment), ΔJ (the differences of relative variable fluorescence at t = 2 ms reference control treatment), ΔI (the differences of relative variable fluorescence at t = 30 ms reference control treatment), and ΔL (the differences of relative variable fluorescence at t = 150 μs reference control treatment) were further analyzed. In addition, we calculated the following fluorescence parameters: *F*_o_ (minimum fluorescence at t = 20 μs), *F*_m_ (maximum fluorescence at P-step), *F*_v_ (variable fluorescence), *F*_v_/*F*_o_ (maximum primary yield of the photochemistry of PSII), *F*_o_/*F*_m_ (*DI*_o_/*ABS* or *φD*_o_, quantum yield at t = 0 for energy dissipation), *F*_v_/*F*_m_ (*TR*_o_/*ABS* or *φP*_o_, maximum quantum yield of primary photochemistry at t = 0), *M*_o_ (approximated initial slope of the fluorescence transient *V* = *F*_t_), *ABS*/*RC* (absorption flux per reaction center), *DI*_o_/*RC* (dissipated energy flux per reaction center at t = 0), *φE*_o_ (*ET*_o_/*ABS*, quantum yield for electron transport at t = 0), *φR*_o_ (*RE*_o_/*ABS*, quantum yield for the reduction of end acceptors of PSI per photon absorbed), and *PI*_abs,total_ (total performance index, measuring the performance up to the PSI end electron acceptors) [9, 31].

### Measurement of free amino acid (FAA), soluble protein and malondialdehyde (MDA) contents

Soluble proteins were extracted from the fresh root and leaf samples using 10% glacial acetic acid and measured using the Coomassie brilliant blue method. The total FAA contents of roots and leves were determined using the ninhydrin method as described by Li et al. [27] and Chen et al. [29]. After extraction with 80% ethanol, the MDA contents of roots and leaves were determined using the thiobarbiturate (TBA) method.

### Measurement of antioxidant enzyme

The frozen root and leaf samples were used for the enzyme assays. After extraction with 50 mmol L^−1^ KH_2_PO_4_-KOH (pH 7.5) containing 0.1 mmol L^−1^ ethylene diamine tetraacetic acid disodium salt (EDTA), 0.3% (w/v) Triton X-100, and 4% (w/v) insoluble polyvinylpolypyrrolidone (PVPP), the activities of the antioxidant enzymes catalase (CAT), peroxidase (POD), and superoxide dismutase (SOD) were determined as previously described [28, 31].

### Statistical analysis

All data were analyzed by one-way variance analysis (ANOVA) using SAS 9.3 (SAS Institute, Cary, NC, USA). Mean values were compared by the least significant difference (LSD) test with the threshold for statistical significance set at *P* < 0.05. Principal component analysis (PCA) was performed to evaluate differences in the growth and physiological characteristics of the branch, upper and lower leaves between the Mg treatment groups using SPSS 22 (IBM, Armonk, NY, USA).

## Results

### Mg deficiency inhibited both parent and lateral plant growth

**Fig. 1:**
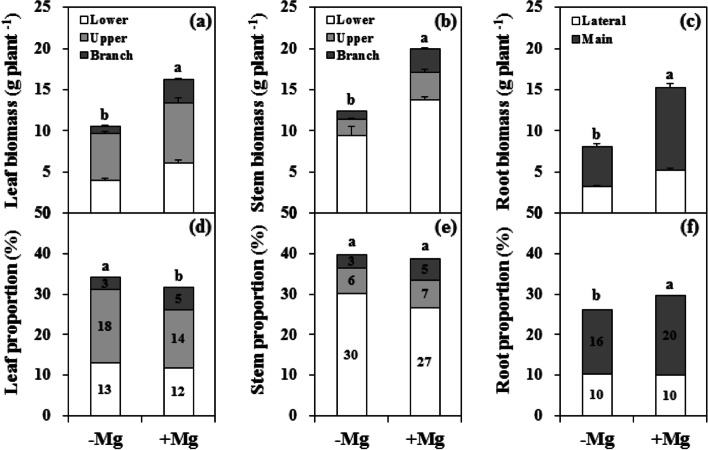
Biomass dry weight (a-c) and distribution (d-f) in leaf (a, d), stem (b, e), and root (c, f) organs of citrus seedlings grown under conditions of Mg deficiency (-Mg) and sufficiency (+ Mg). Leaf and stem biomasses were divided into the branch, upper and lower parts. Root biomass was divided into the main and lateral parts. Data are presented as mean ± standard deviation (*n* = 5). Different letters represent significant differences between the Mg treatment groups at *P* < 0.05

Compared with Mg sufficiency, Mg deficiency markedly inhibited citrus plant growth, with typical symptoms of chlorosis between veins in the LL (Fig. 1; Additional file 1: Fig. S1). The decrease in root biomass (46.8%) was greater than that of the stem (37.9%) and leaf (34.9%). The biomass of the parent and branch leaves was reduced by 28.3% and 66.2%, respectively, while the stem biomass was reduced by 31.5% and 64.5%. The reduction of MR biomass was greater than that of the LR. Also, Mg deficiency altered the biomass distribution between organs, with the proportion increased in the leaf and decreased in the root. However, there was no difference in the biomass of the stems between the two groups.

In terms of plant growth characteristics, the plant height, PL number, number of branches, branch length, and BL number in the Mg deficiency group were reduced by 4.8%, 9.0%, 24.4%, 37.1%, and 24.8%, respectively, while the first branch height was increased by 47.3% (Additional file 1: Fig. S2).

### Mg deficiency altered leaf characteristics and reduced root morphogenesis

**Fig. 2:**
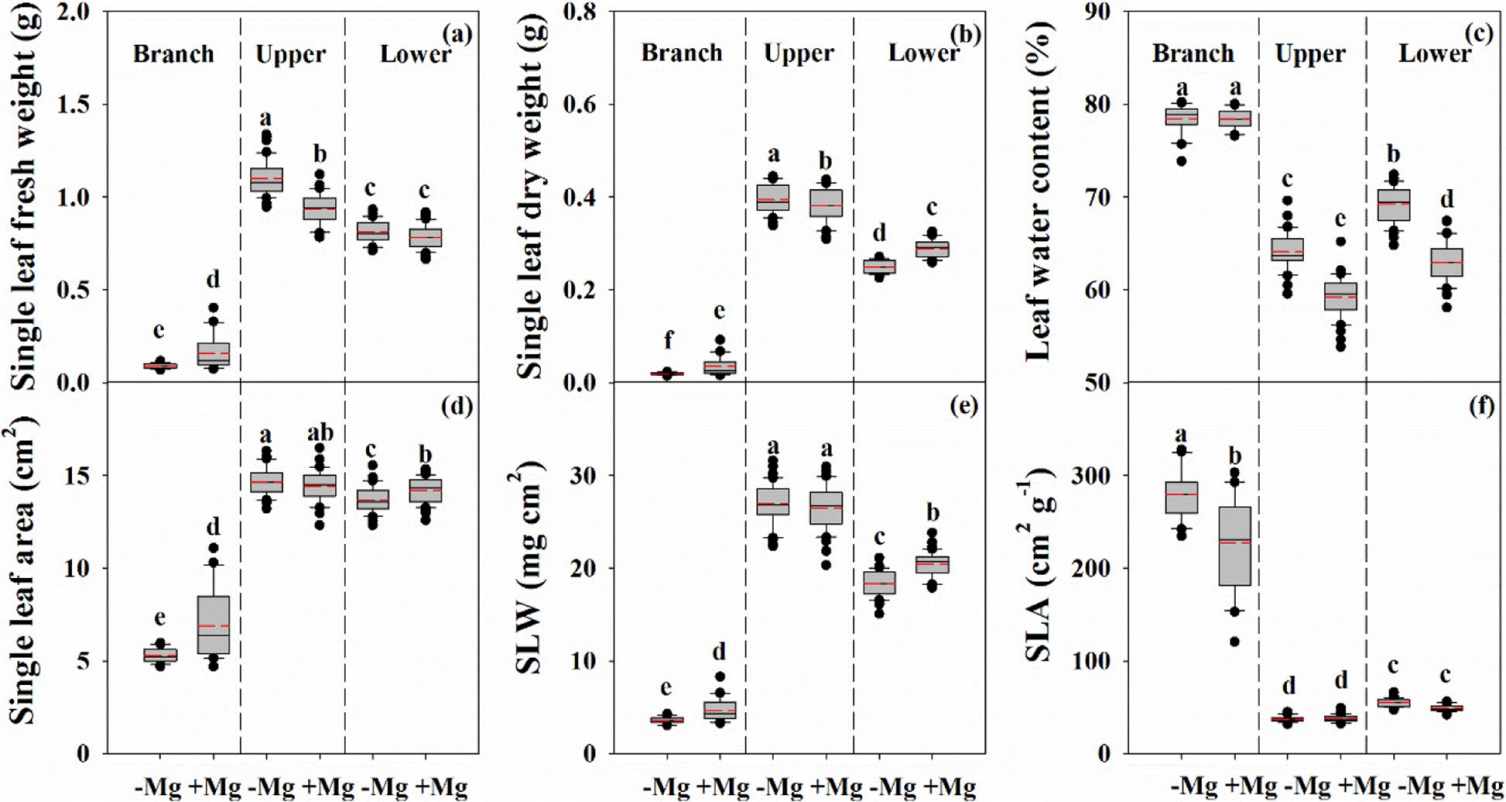
Characteristics of single leaf fresh weight (a), dry weight (b), leaf water content (c), leaf area (d), specific leaf weight (SLW, e) and specific leaf area (SLA, f) in citrus seedlings grown under conditions of Mg deficiency (-Mg) and sufficiency (+ Mg). Data are presented as mean ± standard deviation (*n* = 40). Different letters represent significant differences among organs between the Mg treatment groups at *P* < 0.05

Compared with the Mg sufficiency group, the single leaf fresh weight, dry weight, and water content were increased in the UL in the Mg deficiency group (Fig. 2). In contrast, the single leaf dry weight, leaf area, and SLW of the LL were reduced, while the leaf water content was increased. However, the single leaf fresh weight, dry weight, area, and SLW of the BL in the Mg deficiency group were lower than those in the Mg sufficiency group, while the SLA was increased.

**Fig. 3:**
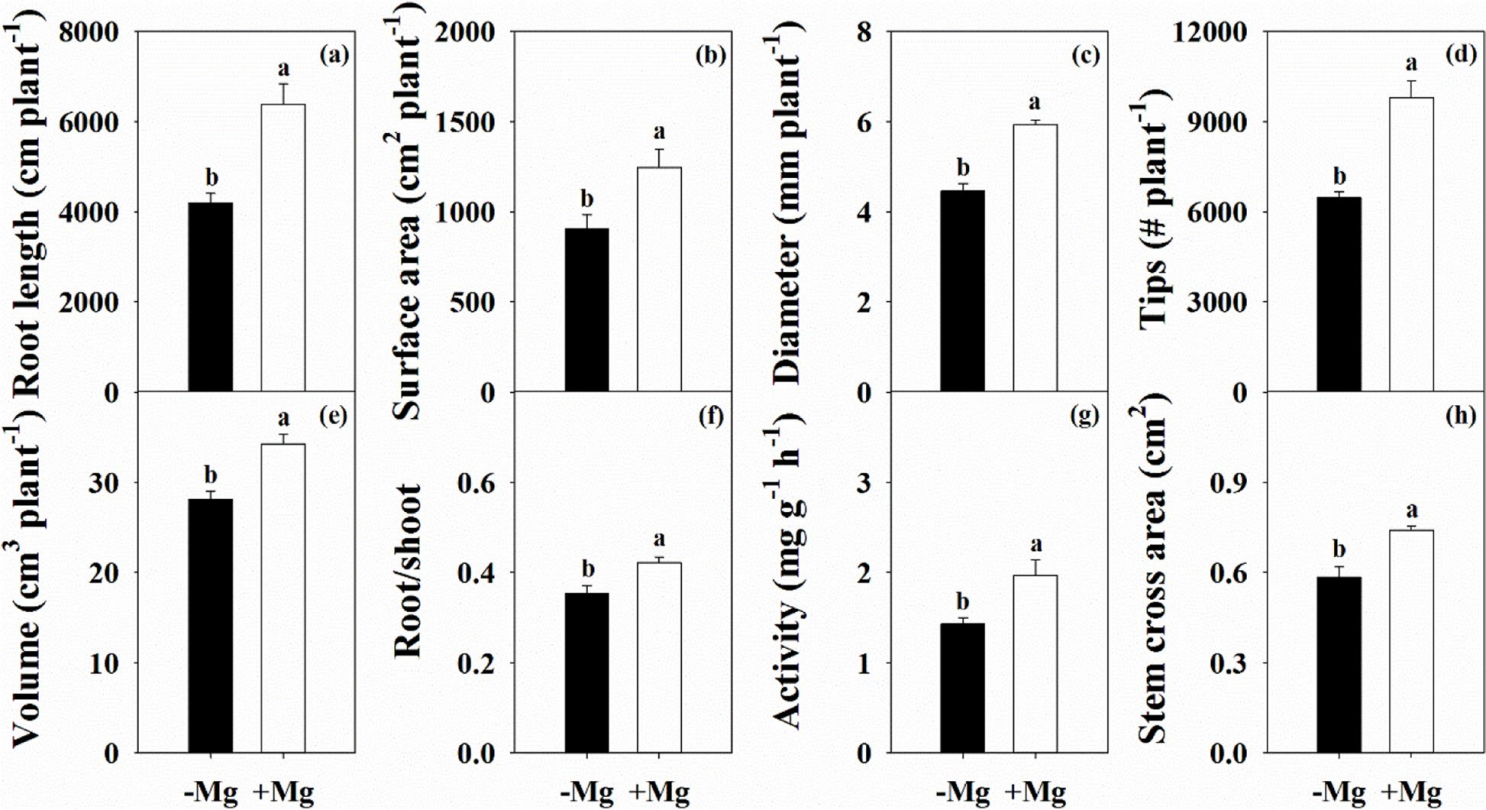
Characteristics of root length (a), surface area (b), average diameter (c), number of tips (d), volume (e), root to shoot ratio (f), activity (g) and stem cross area (h) in citrus seedlings grown under conditions of Mg deficiency (-Mg) and sufficiency (+ Mg). Data are presented as mean ± standard deviation (*n* = 5). Different letters represent significant differences among organs between the Mg treatment groups at *P* < 0.05

Following the changes in leaf growth variables, Mg deficiency also significantly affected root morphological parameters, including length, surface area, average diameter, number of root tips, and volume, which were reduced by 34.3%, 27.2%, 24.6%, 34.0%, and 18.0%, respectively (Fig. 3). Also, the root/shoot ratio, root activity, and stem cross area were decreased by 16.0%, 27.3%, and 21.2%, respectively.

### Mg deficiency reduced Mg uptake and altered Mg distribution

**Fig. 4:**
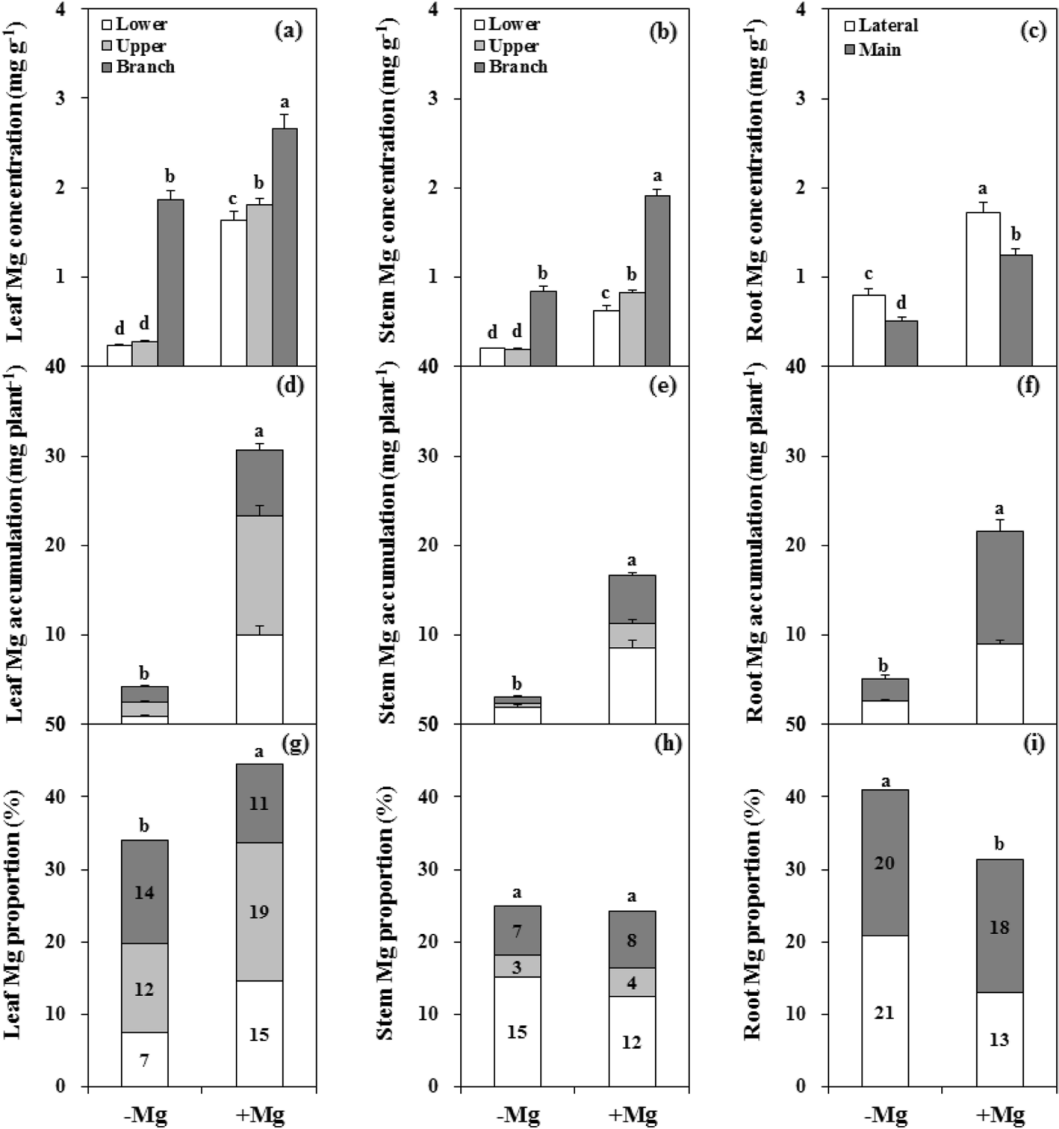
Mg concentration (a-c), accumulation (d-f) and distribution (g-i) in leaf (a, d, g), stem (b, e, h), and root (c, f, i) organs of citrus seedlings grown under conditions of Mg deficiency (-Mg) and sufficiency (+ Mg). Leaf and stem biomasses were divided into the branch, upper and lower parts. Root biomass was divided into main and lateral parts. Data are presented as mean ± standard deviation (*n* = 5). Different letters represent significant differences between the Mg treatment groups at *P* < 0.05

Mg deficient conditions significantly decreased the Mg concentration in different tissues of citrus plants (Fig. 4a-c). In the parent tissues, the Mg concentration was decreased by 85.5% in the leaves and 72.5% in the stem. In the branch tissues, the Mg concentration was decreased by 29.7% in the leaves and 55.7% in the stem. In addition, the Mg concentration was decreased by more than 50% in both the MR (59.1%) and LR (53.5%). However, regardless of the Mg treatment conditions, the Mg concentrations in both the leaf and stem of the branch tissues were markedly higher than those in the parent tissues, and the Mg concentration in the LR was consistently higher than that in the MR.

Under Mg deficient conditions, total Mg accumulation in the plant was reduced by 82.0% (Fig. 4d-f). Mg accumulation in the root, stem, and leaf organs decreased by 76.4%, 81.5%, and 86.3%, respectively, and the decrease in the branch tissues (79.6%) was less than that in the parent tissues (86.4%). Furthermore, in terms of Mg distribution, the proportion was increased in root and decreased in leaf, while there was no difference the proportion in the stem compared with plants grown under Mg sufficient conditions. The proportions of Mg in the PL decreased and increased the LR (Fig. 4g-i).

### Mg deficiency inhibited leaf photosynthesis capacity

**Fig. 5:**
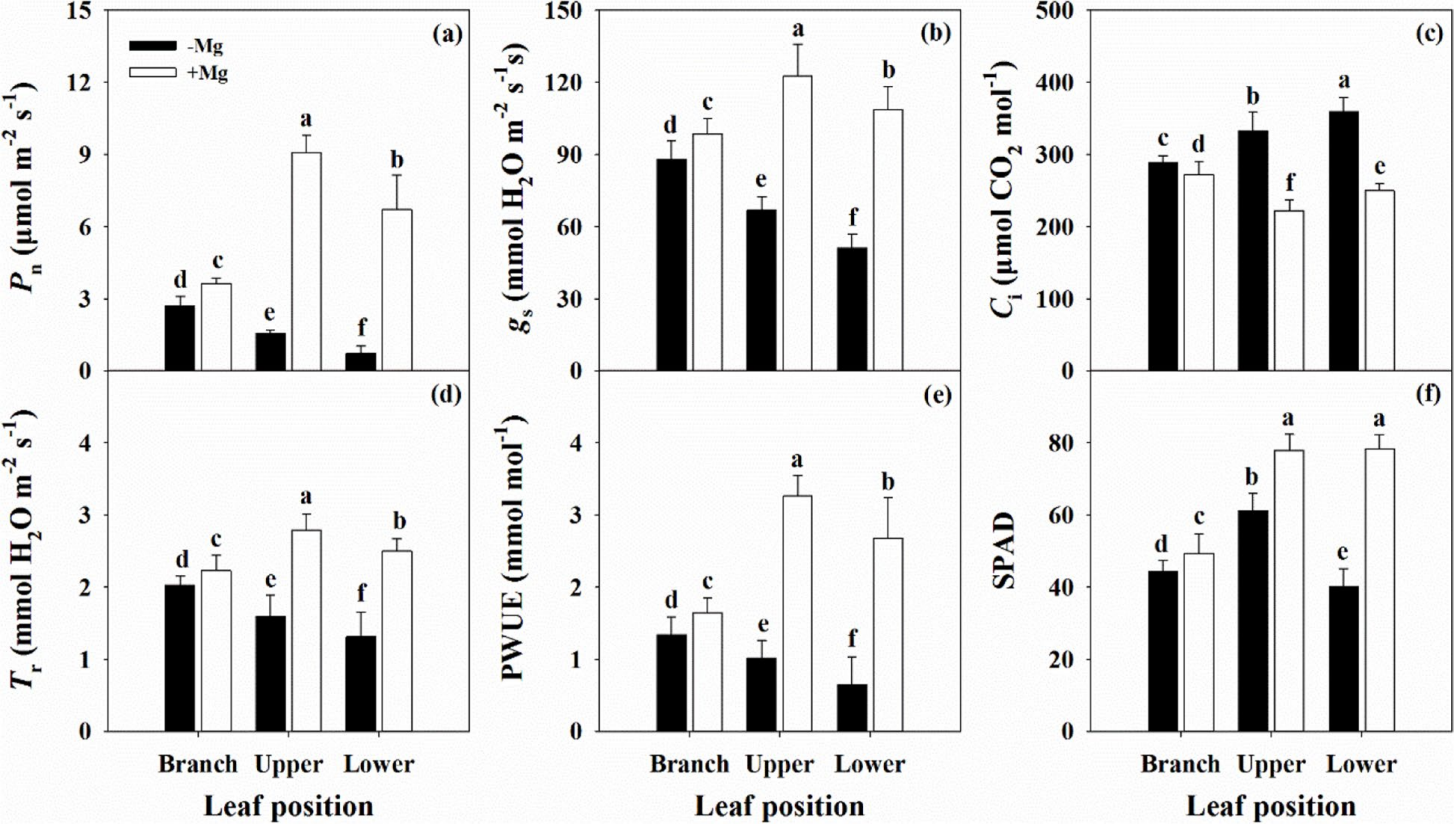
Characteristics of leaf net photosynthetic rate (*P*_n_, a), stomatal conductance (*g*_s_, b), intercellular CO_2_ concentration (*C*_i_, c), transpiration rate (*T*_r_, d), photosynthetic water-use efficiency (PWUE, e) and SPAD value (f, *n* = 40) in citrus seedlings grown under conditions of Mg deficiency (-Mg) and sufficiency (+ Mg). Data are presented as mean ± standard deviation (*n* = 13). Different letters represent significant differences among organs between the Mg treatment groups at *P* < 0.05

The parameters of *P*_n_, *g*_s_, *T*_r_, and PWUE were decreased by Mg deficiency, while *C*_i_ was increased, and the changes intensified as the leaf position decreased (Fig. 5). A similar trend was observed for the SPAD of the chlorophyll pigments (Additional file 1: Fig. S3). The Chl a, Chl b, Chl a + b, and Car contents were reduced across the leaf positions under Mg deficiency, with an increased Chl/Car in the BL and UL, but decreased Chl/Car in the LL.

**Fig. 6:**
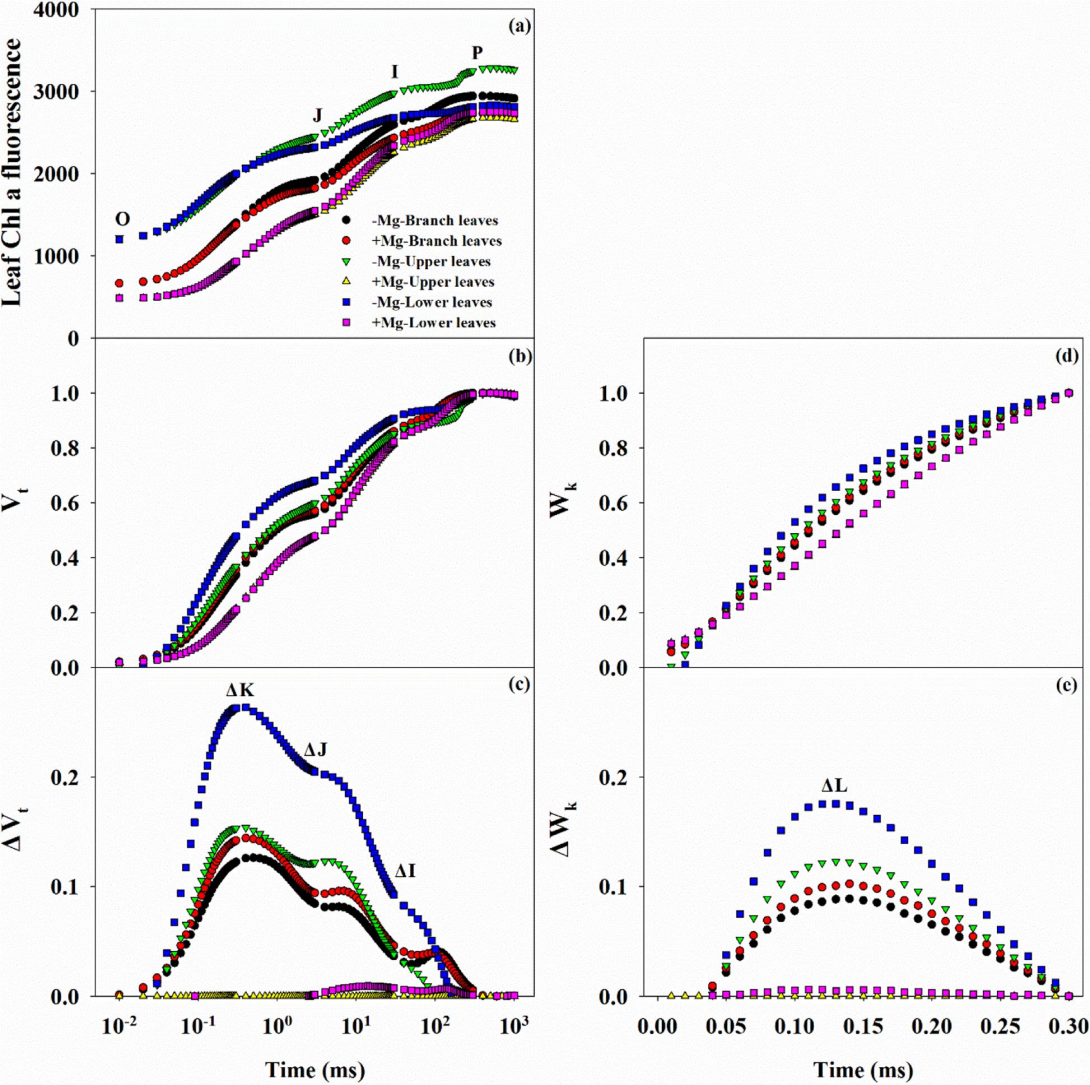
Characteristics of leaf Chl a fluorescence transients OJIP curves (**a**), V_t_ (**b**), ΔV_t_ (**c**) W_k_ (**d**), ΔW_k_ (**e**) in citrus seedlings grown under conditions of Mg deficiency (-Mg) and sufficiency (+ Mg). Data are presented as the mean (*n* = 14)

Changes in the leaf Chl a fluorescence transients may contribute to alterations in gas exchange (Fig. 6). Generally, there were three types of OJIP curves, with the highest levels observed in PL under Mg deficiency, followed by the BL across the Mg treatment conditions, and the lowest levels in the PL under Mg sufficiency (Fig. 6a). Similar trends were also observed in the curves of V_t_ (or ΔV_t)_ and W_k_ (or ΔW_k_), with positive ΔK-, ΔJ-, ΔI-, and ΔL-bands in leaves, especially the LL, under Mg deficiency (Fig. 6b-d).

Analysis of 14 fluorescence parameters showed that the *F*_o_, *F*_o_/*F*_m_, *M*_o_, *ABS*/*RC*, and *DI*_o_*/RC* increased as the leaf position lowered under Mg deficiency, while the opposite trend was observed for *F*_v_, *F*_v_/*F*_o_, *F*_v_/*F*_m_, *φE*_o_, and *φR*_o_ (Additional file 1: Fig. S4). However, with the exception of the *F*_v_, there was no significant difference in any of the parameters in the BL between the Mg treatments. Finally, under conditions of Mg deficiency, the *PI*_abs,total_ was decreased across leaf positions, with a greater reduction in the PL than that in the BL.

### Mg deficiency changed related physiological parameters

**Fig. 7:**
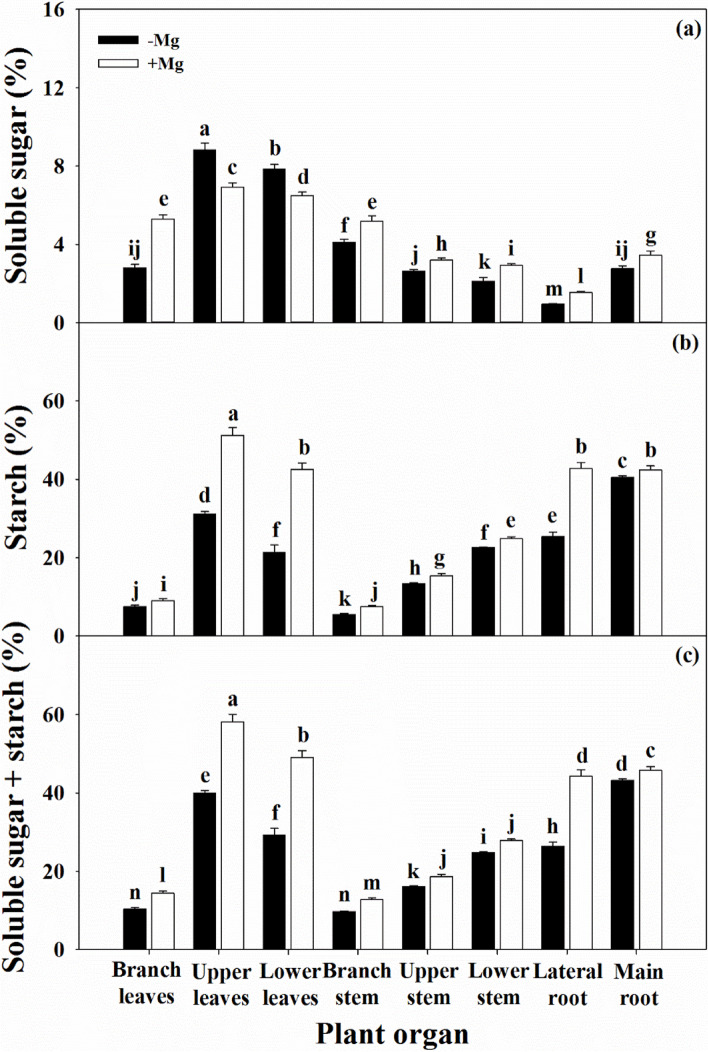
Characteristics of soluble sugar (a), starch (b), and non-structure carbohydrates (soluble sugar + starch, c) in citrus seedlings grown under conditions of Mg deficiency (-Mg) and sufficiency (+ Mg). Data are presented as mean ± standard deviation (*n* = 5). Different letters represent significant differences among organs between the Mg treatment groups at *P* < 0.05

The reduction in the photosynthetic performance of leaves under Mg deficiency will inevitably lead to changes in assimilate production and transport to various organs (Fig. 7). With the exception of the soluble sugar content in the PL, the soluble sugar and starch contents in all tissues were significantly decreased under Mg deficiency compared with those under Mg sufficiency, resulting in reduced NSC content.

**Fig. 8:**
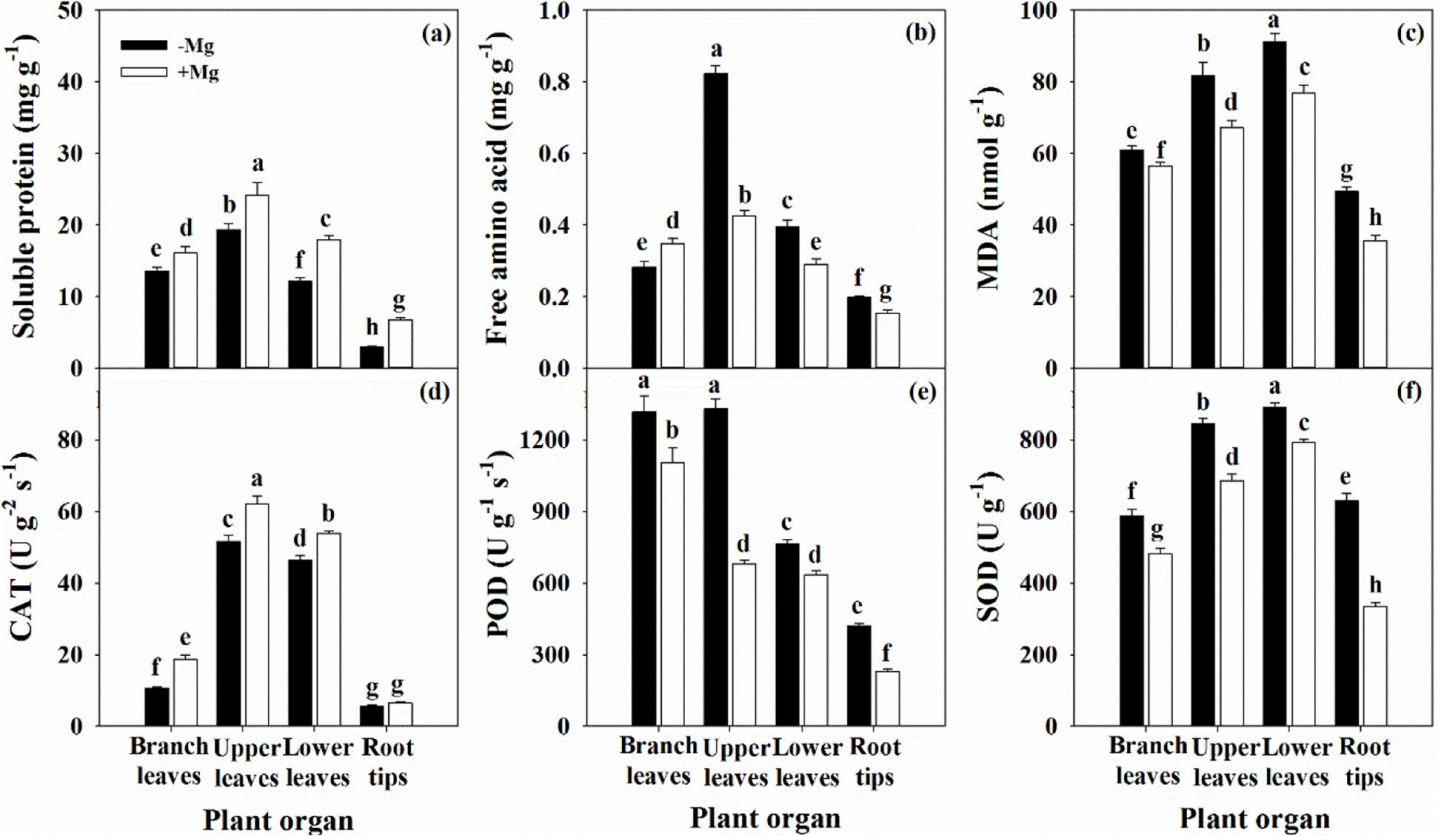
Characteristics of soluble protein (a), free amino acid (b), MDA (c), CAT (d), POD (e) and SOD (f) in citrus seedlings grown under conditions of Mg deficiency (-Mg) and sufficiency (+ Mg). Data are presented as mean ± standard deviation (*n* = 5). Different letters represent significant differences among organs between the Mg treatment groups at *P* < 0.05

The biosynthesis of soluble proteins in test organs was significantly decreased by Mg deficiency (Fig. 8a), which was in contrast to the effects on FAA content, except in the BL (Fig. 8b). In addition, Mg deficiency induced peroxidation of membrane lipids, which was associated with dramatic accumulation of MDA in the leaf and root organs (Fig. 8c). POD and SOD activity was also increased under Mg deficiency, while CAT activity was decreased (Fig. 8d-f).

**Fig. 9:**
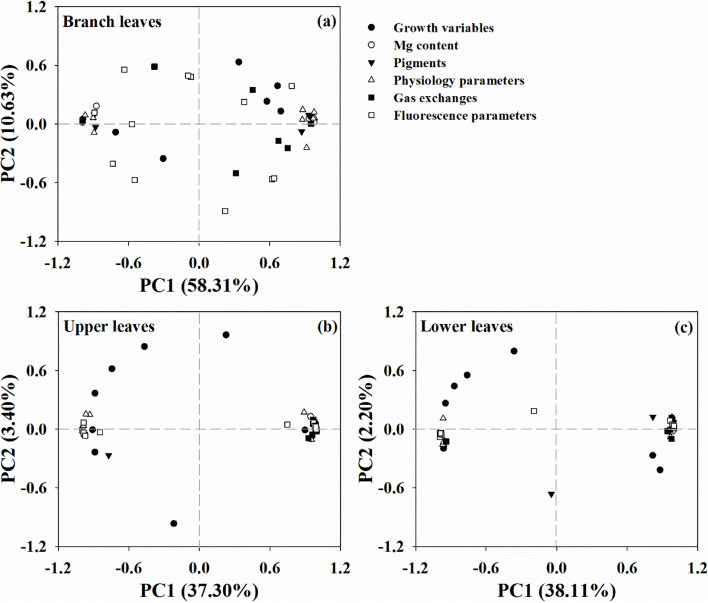
Principal components analysis of the branch leaves (BL (**a**)), upper leaves (UL (**b**)), and lower leaves (LL (**c**)) based on morphological and physiological parameters of citrus seedlings grown under conditions of Mg deficiency (-Mg) and sufficiency (+ Mg)

Moreover, PCA of the differences between the Mg treatment groups showed that the total variations were highly separated in the BL, but highly clustered in the PL (Fig. 9). The first two components accounted for 68.9% (58.3% for PC1 and 10.6% for PC2), 40.7% (37.3% for PC1 and 3.4% for PC2), and 40.3% (38.1% for PC1 and 2.2% for PC2) of the total variation in the BL, UL, and LL, respectively, and explained the significantly higher variation in the BL compared with that in the PL.

## Discussion

### Mg deficiency inhibits plant growth and induces changes in morphology and biomass partitioning

Citrus plants are characterized by low Mg sensitivity and a high Mg demand [9, 25, 27]. Mg deficiency can affect physiological and biochemical processes, thus eventually influencing plant growth, yield, and fruit quality [19, 22, 26]. Our study showed that citrus plant biomass was depressed by Mg deficiency, with greater inhibition of root than shoot (Fig. 1; Additional file 1: Fig. S1), resulting in a lower root/shoot ratio (Fig. 3f). This change reflects the biomass allocation and adaptability of citrus plants under stress conditions [3–5, 33]. Previous studies have yielded inconsistent results in terms of the effects of Mg deficiency on root/shoot ratio, with a decreased ratio reported in banana [11, 34] and citrus [35], whereas increased ratios have been reported in rice [36], maize [37], sugar beet [10, 12], and *Arabidopsis thaliana* [33]. A meta-analysis by Hauer-Jákli and Tränkner [5] revealed that Mg deficiency had no effect on the root/shoot ratio, while Mg sufficiency had a more positive effect on root biomass than on shoot biomass. The effects of Mg deficiency on the root/shoot ratio of citrus plants observed in this study are inconsistent with previous reports [27, 35, 38], and dynamic changes in the root/shoot have also been observed in rice [39]. These results suggest that the adaptive strategies of biomass allocation in response to Mg deficiency may be affected by factors such as plant species, pre-treatment, Mg dosage, culture time, and growth environment [5, 10, 11, 39]. Furthermore, our results showed that Mg deficiency had a greater inhibitory effect on branch tissue growth than that on parent tissue (Fig. 1), which highlights the physiological function of Mg nutrition in woody plants.

The root is the main organ of nutrient and water uptake and its morphological and functional characteristics reflect the tolerance of plants to environmental stress conditions, including nutrient stress [33]. Our results showed that Mg deficiency induced a reduction of all root morphological parameters in citrus plants (Fig. 3). In particular, there was a much greater decline in the biomass of the MR compared with that of the LR (Fig. 1), which will inevitably affect the shoot properties, and inhibit branch growth (Additional file 1: Fig. S2). In leaf, reduced dry weight was associated with reduced area, resulting in a lower SLW (Fig. 2). Interestingly, the leaf water content under Mg deficiency was higher than that under Mg sufficiency in the parent, while there was no effect on the branch (Fig. 2). These findings indicate that Mg deficiency modulates the water cycle properties in citrus plants, which accounts for the lower PWUE (Fig. 5e). A relationship between Mg supply and water uptake was reported by Tränkner et al. [40], who found that Mg deficiency decreased the total biomass and shoot WUE, but increased the leaf WUE in barley plants. These results explained the reduction in plant biomass of different organs of the parent and branch tissues, and changed its allocation under Mg deficiency conditions. These changes further reflect the negative effects of Mg deficiency on the physiological and ecological functions of citrus plants.

### Mg deficiency reduces plant Mg uptake but promotes Mg transport from old organs to new

Previous studies have demonstrated that plant growth and Mg uptake is affected by Mg supply in many plants, including cereals [13, 37], vegetables [10, 12, 41], fruit [9, 33], and other plants [42, 43]; however, the mechanisms of Mg transport and distribution in plants under Mg deficiency remain poorly understood, especially in parent and branch tissues of citrus plants. In the present study, citrus plants under Mg deficiency displayed much lower Mg concentrations and accumulation in all organs, with the Mg proportion increased in leaf and decreased in root, but with no effect on the stem compared with plants grown under Mg sufficient conditions (Fig. 4). Surprisingly, the Mg concentrations in both the leaf and stem of branch tissues were dramatically higher than those in the parent regardless of the Mg conditions, while the Mg concentration was higher in the LR than that in the MR (Fig. 4). These results imply that there is greater mobility of Mg in citrus plants from parent to branch, and the higher Mg concentrations in the branch and LR may be related to maintenance of their physiological functions.

It is generally accepted that the Mg remobilization ability of plants determines the adaptation response to Mg deficiency [10, 44]. Ye et al. [9] reported that leaf Mg concentration decreased with increasing leaf age in citrus plants under Mg deficiency condition, and similar observations were reported by Li et al. [27]. Compared with Mg sufficiency, the Mg concentration in branch organs relative to that Mg in the parent organs was improved under Mg deficiency (Fig. 4). These results further revealed the high Mg transport capacity of citrus plants, with Mg remobilization from parent to branch organs enhanced under Mg deficiency. In accordance with this, He et al. [33] reported that Mg deficiency decreased the Mg concentration in all tested tissues of banana plants, and the Mg transport capacity from old leaves to new increased with the duration of the cultivation period. However, our findings highlight the influence of the capacity for Mg transport from the parent to branch tissues on the responses to Mg deficiency in citrus plants. This phenomenon accounts for the alterations in photosynthesis and related physiological parameters induced by Mg deficiency in different parent and branch organ tissues.

### Mg deficiency damages the photosynthetic system and induces relative metabolic disorders

Photosynthesis is responsible for the production of materials and energy in plants. This physiological processes is highly sensitive to environmental factors such as Mg status [2–4, 12]. Generally, Mg deficiency limits photosynthesis and contributes to reduced plant growth, mainly through its ability to affect CO_2_ fixation, transportation and the distribution of photo-assimilates. This is consistent with our results showing that gas exchange parameters, including *P*_n_, *g*_s_, and *T*_r_, were significantly decreased, while *C*_i_ was increased, especially in parent leaves (Fig. 5). Similar results have been reported in broad beans [41], indicating that the lower *P*_n_ in Mg deficient leaves may be affected by stomatal factors. In contrast, Hermans et al. [12] revealed that Mg deficiency changed the physiological functions of PSI and PSII by reducing the Chl a and b contents. This was also supported by Yang et al. [31] and Ye et al. [9], who reported that Mg deficiency induced alterations in photosynthetic electron transport capacity in citrus leaves. These results suggested that the decreased leaf CO_2_ assimilation induced by Mg deficiency was associated with stomatal and non-stomatal factors. Our results showed that Mg deficiency decreased the Chl content and changed Chl fluorescence parameters in leaf tissues (Fig. 6; Additional file 1: Figs. S3 and S4), which indicated lower energy exchange and transfer or high energy excitation of PSII, and reduction of electron transport acceptors in PSI [9, 18, 31]. Mg deficiency induced increases in *F*_o_ and *F*_m_ and decreased *F*_v_, resulting in decreased *F*_v_/*F*_o_ and *F*_v_/*F*_m_ ratios (Additional file 1: Fig. S4). These findings indicate that Mg deficiency disrupts the thylakoid structure, while inducing photo-inhibition and photo-oxidative damage. In short, the whole photosynthetic electron transport chain from the donor side of PSII to the acceptor side of PSI might be responsible for the decline in CO_2_ assimilation in Mg deficient leaves [3, 4, 12], with little effect on branch leaves. However, Tränkner and Jaghdani [45] reported that the critical Mg concentration required for biomass and yield formation was higher than that for photosynthetic processes in wheat and sunflower plants, and revealed that Mg deficiency induces photo-oxidative stress by limiting CO_2_ assimilation but not by limiting photosynthetic light utilization [46].

Mg deficiency reduces the biosynthesis of photosynthetic products, while inducing the accumulation of NSC in the leaves of many plants, such as sugar beet [10], coffee [43], wheat [13], and banana [33], thus accounting for the ability of Mg deficiency to limit the transport of assimilates from source to sink. Furthermore, higher soluble sugar content in leaves may also inhibit photosynthesis via a feedback mechanism under Mg deficiency [3, 12, 38]. In the present study, except for soluble sugars content of PL was increased, Mg deficiency decreased the NSC contents of both soluble sugar and starch in different organs (Fig. 7). These findings explain the combined effect of long-term Mg deficiency on the growth of secondary organs in citrus plants. A similar result was also reported in Mg deficient citrus orchards [18]. In contrast, higher NSC accumulation in the leaves of plants with lower NSC content in the roots was also consistently observed under Mg deficiency in sugar beet [10], citrus [27, 31], and banana [34] plants. However, the relationship between Mg nutrition status and photosynthesis or carbohydrate transportation is controversial, Hermans et al. [10] revealed that Mg deficiency enhanced the expression of the *BvSUT1* gene that encodes a companion of the sucrose/H^+^ symporter, but did not affect sucrose loading into the phloem, resulting in sucrose accumulation in leaves. Moreover, anatomic investigations revealed that Mg deficiency induced cell wall lignification in the vascular cambium and spongy parenchyma of leaves, thus affecting which leaf assimilate transport in citrus plants [38].

With the exception of the FAA content in branch leaves, the soluble protein content decreased and the FAA content increased across all organs (Fig. 8a,b). A similar result was observed by Jin et al. [47], who found the soluble protein was significantly decreased in leaf, but not in root under Mg deficiency. Li et al. [27] reported that the soluble protein and FAA contents in leaf were decreased by Mg deficiency, especially in the LL. Furthermore, the activity of the NR, GS, and GOGAT enzymes required for N metabolism in root and leaf were all inhibited; this finding also supported by Ding et al. [36]. Ye et al. [9] showed that the N concentration in the leaf blade, but not in the leaf vein, was reduced with increasing leaf age in citrus plants under Mg deficiency. In contrast, Jezek et al. [37] found that the capacity for GS-mediated glutamine synthesis from FAA and glutamate in leaves was not impaired by Mg deficiency, demonstrating that a reduction in the protein content is not inevitable under these conditions. These results indicated that Mg deficiency disrupts N uptake and metabolism in citrus plants, and was supported by the positive interactions between N and Mg observed in lemon trees [16] and forage plants [48]. Furthermore, the mechanism by which Mg supports N uptake has been confirmed in soybean [49]. Huang et al. [38] and Ye et al. [9] also reported that Mg deficiency alters the concentrations of other nutrients in citrus plants.

It is worth mentioning that *P*_n_ decreased with increasing MDA content in leaf (Fig. 8c), indicating that the changes in the chloroplast membrane lipids might be related to the decreased CO_2_ assimilation induced by Mg deficiency [28, 31]. Also, the higher MDA content in root reflects the reduction in physiological functions, such as root activity (Fig. 3g). Interestingly, the antioxidant system was not always impaired under Mg deficiency in this study, in which we observed increased activity of the antioxidant enzymes POD and SOD, while CAT activity was decreased across the leaf and root organs (Fig. 8d-f). Tang et al. [18] reported that Mg deficient leaves in citrus plants have an adaptive ability to avoid photo-oxidative damage by up-regulation of antioxidant metabolism; this finding was also supported by Cakmak and Kirkby [3] and Farhat et al. [4]. Shang and Feierabend [50] reported that CAT is sensitive to photo-inactivation, leading to oxidative damage caused by the limited capacity for light utilization by the chloroplast. This process that can be induced or exacerbated by Mg deficiency [28, 31, 42]. Furthermore, combined with the passive effect of Mg deficiency on photosynthetic electron transport (Fig. 6; Additional file 1: Fig. S4), this phenomenon indicates that the antioxidant system is unable to cope with the photo-oxidation damaged by Mg deficiency. These results further support the concept that Mg deficiency induces disruption of the antioxidant system in citrus plants [18, 28, 31]. Similar results have also reported in other plants, such as mulberry [42], and coffee [43].

A comprehensive analysis also revealed variations in the morphological and physiological parameters of the leaves at different positions. The variations were found to be significantly separated between the parent and branch leaves, with a higher degree of clustering in the branch leaves than that in the parent leaves (Fig. 9). A similar result was observed by Ye et al. [9], who found that variations in photosynthetic parameters clustered with increasing leaf age in Mg deficient citrus plants. These findings indicated that the high level of separation of branch leaves might be related to the difference in Mg concentration between the two Mg treatments, which further implied that the high Mg remobilization from parent to branch contributed to the differences in leaf positions. The molecular mechanism of the response of citrus plants to Mg deficiency has been gradually revealed with advances in research. Using the cDNA-AFLP method, Jin et al. [47] reported that Mg deficiency induced differential expression of 71 and 70 genes in root and leaf, respectively, while Yang et al. [35] identified a total of 4,864 differentially expressed genes in Mg deficient leaves using the RNA-Seq method. Liang et al. [51] reported 170 differentially expressed microRNAs (101 up-regulated and 69 down-regulated) in root tissues under Mg deficiency, while 146 (75 up-regulated and 71 down-regulated) were found in leaf tissues [52]. Peng et al. [53] also identified 90 differentially expressed proteins (59 up-regulated and 31 down-regulated) in leaf tissues under Mg deficient conditions, while 31 (19 up-regulated and 12 down-regulated) were identified in root tissues. These differentially expressed genes, microRNAs, and proteins functioned mainly in carbohydrate and energy metabolism, protein metabolism, cell wall and cytoskeleton metabolism, nucleic acid metabolism, lipid metabolism, cell transport, stress responses, and the antioxidant system [35, 47, 51–53]. Metabolomics further revealed that Mg deficiency increased the abundance of lipids and lipid-like molecules, but decreased the abundance of phenylpropanoids and polyketides in citrus leaves [54]. Moreover, Hermans et al. [55] identified a relationship between Mg deficiency and the leaf circadian clock, while Li et al. [15] revealed the process and underlying mechanism by which the *OsMGT3* transporter is regulated by Mg fluctuations in chloroplasts and contributes to photosynthesis in rice.

## Conclusions

In the present study, we report for the first time the effects of Mg deficiency on branch growth in citrus seedlings. Mg deficiency directly inhibited Mg uptake and altered its transport and mobility from parent to branch in citrus plants. This then reduced photosynthetic productivity by decreasing the levels of leaf pigments and impairing the photosynthetic electron transport chain. Furthermore, Mg deficiency indirectly induced related physiological disorders, finally disrupting the balance between the parent and branch tissues. PCA also revealed a high degree of separation in the variation of branch leaves, and a high degree of clustering in the variation of parent leaves. These findings provide support for the concept that the Mg supply status influences citrus plant growth by regulating Mg uptake and physiological metabolism. In conclusion, our results reveal a positive relationship between Mg remobilization and lateral organ growth, which is helpful in developing Mg management strategies to improve citrus production.

## Supplementary Information

**Additional file 1: Figure S1.** Growth characteristics and symptoms of citrus seedlings grown under conditions of Mg deficiency (-Mg) and sufficiency (+Mg). **Figure S2.** Characteristics of plant height (a), number of plant leaves (b), first branch height (c), number of branches (d), branch length (e) and number of branch leaves (f) in citrus seedlings grown under conditions of Mg deficiency (-Mg) and sufficiency (+Mg). Data are presented as mean ± standard deviation (*n* = 25). Different letters represent significant differences among organs between the Mg treatment groups at *P* < 0.05. **Figure S3.** Characteristics of leaf Chl a (a), Chl b (b), Car (c), Chl a+b (d), Chl a/b (e) and Chl/Car (f) in citrus seedlings grown under conditions of Mg deficiency (-Mg) and sufficiency (+Mg). Data are presented as mean ± standard deviation (*n* = 10). Different letters represent significant differences among organs between the Mg treatment groups at *P* < 0.05.** Figure S4.** Characteristics of leaf Chl a fluorescence transient parameters with *F*_o_ (a), *F*_m_ (b), *F*_v_ (c), *F*_v_/*F*_o_ (d), *F*_o_/*F*_m_ (*DI*_o_/*ABS* or *φD*_o_, e), *F*_v_/*F*_m_ (*TR*_o_/*ABS* or *φP*_o_, e), *M*_o_ (g), *ABS*/*RC* (h), *DI*_o_/*RC* (i), *φE*_o_ (*ET*_o_/*ABS*, j), *φR*_o_ (*RE*_o_/*ABS*, k), and *PI*_abs,total_ (l) in citrus seedlings grown under conditions of Mg deficiency (-Mg) and sufficiency (+Mg). Data are presented as mean ± standard deviation (*n* = 14). Different letters represent significant differences among organs between the Mg treatment groups at *P* < 0.05.

## Data Availability

All data sustaining the results in this study are included in this article and its supplementary information files. Other datasets generated or analyzed during this study are available upon reasonable request from the corresponding author (Jiuxin Guo).

## Abbreviations

Mg: Magnesium
-Mg: Mg deficiency
+ Mg: Mg sufficient
MR: Main root
LR: Lateral root
PS: Parent stem
US: Upper stem in PS
LS: Lower stem in PS
BS: Branch stem
PL: Parent leaves
UL: Upper leaves in PL
LL: Lower leaves in PL
BL: Branch leaves
SLW: Specific leaf weight
SLA: Specific leaf area
NSC: Non-structural carbohydrate
Chl: Chlorophyll
Car: Carotenoids
SPAD: Relative chlorophyll content
PPFD: Photosynthetic photon flux density
*P*_n_: Net photosynthetic rate
*g*_s_: Stomatal conductance
*C*_i_: Intercellular CO_2_ concentration
*T*_r_: Transpiration rate
PWUE: Photosynthetic water-use efficiency
OJIP: Chlorophyll a fluorescence transients of dark adaptation
V_t_: Relative variable fluorescence at t between *F*_o_ and *F*_m_
W_k_: Relative variable fluorescence at t between *F*_o_ and *F*_300μ__s_
ΔV_t_: The differences of relative variable fluorescence at t between *F*_o_ and *F*_m_ reference control treatment
ΔW_k_: The differences of relative variable fluorescence at t between *F*_o_ and *F*_300μ__s_ reference control treatment
ΔK: The differences of relative variable fluorescence at t = 300 μs reference control treatment
ΔJ: The differences of relative variable fluorescence at t = 2 ms reference control treatment
ΔI: The differences of relative variable fluorescence at t = 30 ms reference control treatment
ΔL: The differences of relative variable fluorescence at t = 150 μs reference control treatment
*F*_o_: Minimum fluorescence at t = 20 μs
*F*_m_: Maximum fluorescence at P-step
*F*_v_: Variable fluorescence
*F*_v_/*F*_o_: Maximum primary yield of the photochemistry of PSII
*F*_o_/*F*_m_: *DI*_O_/*ABS* or *φD*_o_, quantum yield at t = 0 for energy dissipation
*F*_v_/*F*_m_: *TR*_O_/*ABS* or *φP*_o_, Maximum quantum yield of primary photochemistry at t = 0
*M*_o_: Approximated initial slope of the fluorescence transient V = *F*_t_
*ABS*/*RC*: Absorption flux per reaction center
*DI*_o_/*RC*: Dissipated energy flux per reaction center at t = 0
*φE*_o_: *ET*_O_/*ABS*, quantum yield for electron transport at t = 0
*φR*_o_: *RE*_O_/*ABS*, quantum yield for the reduction of end acceptors of PSI per photon absorbed
*PI*_abs,total_: Total performance index, measuring the performance up to the PSI end electron acceptors
FAA: Free amino acid
MDA: Malondialdehyde
CAT: Catalase
POD: Peroxidase
SOD: Superoxide dismutase
PCA: Principal component analysis
