# Supplementary material for: Differences in morphological and physiological features of citrus seedlings are related to Mg transport from the parent to branch organs
Source: BMC Plant Biol. 2021 May 27;21:239. doi: 10.1186/s12870-021-03028-z (PMC8157678; doi:10.1186/s12870-021-03028-z)
Supplement: Supplementary file 1 — Additional file 1: Figure S1. Growth characteristics and symptoms of citrus seedlings grown under conditions of Mg deficiency (-Mg) and sufficiency (+Mg). Figure S2. Characteristics of plant height (a), number of plant leaves (b), first branch height (c), number of branches (d), branch length (e) and number of branch leaves (f) in citrus seedlings grown under conditions of Mg deficiency (-Mg) and sufficiency (+Mg). Data are presented as mean ± standard deviation (n = 25). Different letters represent significant differences among organs between the Mg treatment groups at P < 0.05. Figure S3. Characteristics of leaf Chl a (a), Chl b (b), Car (c), Chl a+b (d), Chl a/b (e) and Chl/Car (f) in citrus seedlings grown under conditions of Mg deficiency (-Mg) and sufficiency (+Mg). Data are presented as mean ± standard deviation (n = 10). Different letters represent significant differences among organs between the Mg treatment groups at P < 0.05. Figure S4. Characteristics of leaf Chl a fluorescence transient parameters with Fo (a), Fm (b), Fv (c), Fv/Fo (d), Fo/Fm (DIo/ABS or φDo, e), Fv/Fm (TRo/ABS or φPo, e), Mo (g), ABS/RC (h), DIo/RC (i), φEo (ETo/ABS, j), φRo (REo/ABS, k), and PIabs,total (l) in citrus seedlings grown under conditions of Mg deficiency (-Mg) and sufficiency (+Mg). Data are presented as mean ± standard deviation (n = 14). Different letters represent significant differences among organs between the Mg treatment groups at P < 0.05. [file 12870_2021_3028_MOESM1_ESM.docx]

**Supplementary information**

**Differences in morphological and physiological features of citrus seedlings are related to Mg transport from the parent to branch organs**

Yamin Jia^1,2†^, Hao Xu^1,2†^, Yuwen Wang^1,2^, Xin Ye^1^, Ningwei Lai^1^, Zengrong Huang^1^, Lintong Yang^1^, Yan Li^1^, Li-Song Chen^1^, Jiuxin Guo^1,2*^

^1^ Fujian Provincial Key Laboratory of Soil Environmental Health and Regulation, College of Resources and Environment, Fujian Agriculture and Forestry University, Fuzhou 350002, China

^2^ International Magnesium Institute, College of Resources and Environment, Fujian Agriculture and Forestry University, Fuzhou 350002, China

† Yamin Jia and Hao Xu contributed equally to this work.

**^*^ Corresponding author:**

To whom correspondence should be addressed.

Name: Jiuxin Guo

E-mail: jiuxinguo@hotmail.com

Tel: ++86-591-83789361

Address: No.15 Shanxiadian Road, Cangshan District, Fuzhou City, Fujian Province, China 350002


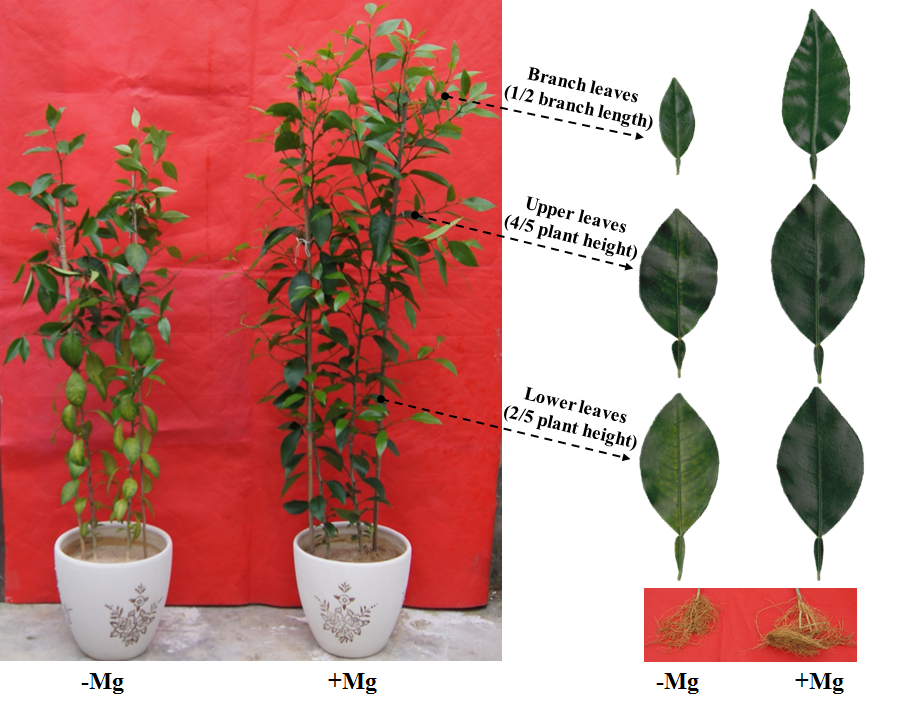


**Fig. S1.** Growth characteristics and symptoms of citrus seedlings grown under conditions of Mg deficiency (-Mg) and sufficiency (+Mg).





**Fig. S2.** Characteristics of plant height (a), number of plant leaves (b), first branch height (c), number of branches (d), branch length (e) and number of branch leaves (f) in citrus seedlings grown under conditions of Mg deficiency (-Mg) and sufficiency (+Mg). Data are presented as mean ± standard deviation (n = 25). Different letters represent significant differences among organs between the Mg treatment groups at *P* < 0.05.

**

**

**Fig. S3.** Characteristics of leaf Chl a (a), Chl b (b), Car (c), Chl a+b (d), Chl a/b (e) and Chl/Car (f) in citrus seedlings grown under conditions of Mg deficiency (-Mg) and sufficiency (+Mg). Data are presented as mean ± standard deviation (n = 10). Different letters represent significant differences among organs between the Mg treatment groups at *P* < 0.05.

**

**

**Fig. S4.** Characteristics of leaf Chl a fluorescence transient parameters with *F*_o_ (a), *F*_m_ (b), *F*_v_ (c), *F*_v_/*F*_o_ (d), *F*_o_/*F*_m_ (*DI*_o_/*ABS* or *φD*_o_, e), *F*_v_/*F*_m_ (*TR*_o_/*ABS* or *φP*_o_, e), *M*_o_ (g), *ABS*/*RC* (h), *DI*_o_/*RC* (i), *φE*_o_ (*ET*_o_/*ABS*, j), *φR*_o_ (*RE*_o_/*ABS*, k), and *PI*_abs,total_ (l) in citrus seedlings grown under conditions of Mg deficiency (-Mg) and sufficiency (+Mg). Data are presented as mean ± standard deviation (n = 14). Different letters represent significant differences among organs between the Mg treatment groups at *P* < 0.05.
